# Polyphenol oxidase affects normal nodule development in red clover (*Trifolium pratense* L.)

**DOI:** 10.3389/fpls.2014.00700

**Published:** 2014-12-17

**Authors:** K. Judith Webb, Alan Cookson, Gordon Allison, Michael L. Sullivan, Ana L. Winters

**Affiliations:** ^1^Institute of Biological, Environmental and Rural Sciences, Aberystwyth UniversityAberystwyth, UK; ^2^US Dairy Forage Research Center, Agricultural Research Service, United States Department of AgricultureMadison, WI, USA

**Keywords:** RNAi transformants, red clover, polyphenol oxidase (PPO), nodule, antioxidant activity, phenolics, Raman microspectrometry

## Abstract

Polyphenol oxidase (PPO) may have multiple functions in tissues depending on its cellular or tissue localization. Here we use PPO RNAi transformants of red clover (*Trifolium pratense*) to determine the role PPO plays in normal development of plants, and especially in N_2_-fixing nodules. In red clover, PPO was not essential for either growth or nodule production, or for nodule function in plants grown under optimal, N-free conditions. However, absence of PPO resulted in a more reduced environment in all tissues, as measured by redox potential, and caused subtle developmental changes in nodules. Leaves and, to a lesser extent nodules, lacking PPO tended to accumulate phenolic compounds. A comparison of nodules of two representative contrasting clones by microscopy revealed that nodules lacking PPO were morphologically and anatomically subtly altered, and that phenolics accumulated in different cells and tissues. Developing nodules lacking PPO were longer, and there were more cell layers within the squashed cell layer (SCL), but the walls of these cells were less thickened and the cells were less squashed. Within the N_2_-fixing zone, bacteroids appeared more granular and were less tightly packed together, and were similar to developmentally compromised bacteroids elicited by catalase mutant rhizobia reported elsewhere.

## INTRODUCTION

Polyphenol oxidases (PPOs) are ubiquitous copper-containing enzymes with antioxidant activity. They catalyze oxidation of diphenols to quinones and cause oxidative browning of plant tissues when subjected to biotic and abiotic stresses (Yoruk and Marshall, 2003; Mayer, 2006). Here, the term PPO refers specifically to monophenolase (tyrosinase; EC 1.14.18.1) and *o*-diphenol oxidase (catechol oxidase; EC 1.10.3.1), which oxidize monophenols to *o*-diphenols and *o*-diphenols to *o-*quinones respectively. These highly reactive quinones polymerize, with themselves or with amino acids and proteins, to produce high molecular weight colored complexes (Walker and Ferrar, 1998).

A broad range of plant species have significant PPO activity (Mayer, 2006) and many show differential expression in cells and tissues (Thygesen et al., 1995; Thipyapong et al., 1997; Shetty et al., 2011; Webb et al., 2013). However, while PPOs catalyze oxidative activity they are generally viewed as part of a plant’s initial defense arsenal, rather than as another aspect of the redox systems deployed during normal plant development.

Evidence is accumulating for PPO’s role in defending plants from biotic stresses using transgenic or mutant plants with over-expressed and suppressed/reduced PPO (Thipyapong et al., 2004, 2007; Winters et al., 2008a). The resistance mechanisms have not been clarified and may vary between species.

Challenging *Lycospersicon esculentum* (tomato) plants with the pathogen *Pseudomonas syringae* has provided convincing direct evidence for PPO’s role in disease resistance: overexpressing PPO increased resistance while suppression of PPO expression using antisense increased plant susceptibility (Thipyapong et al., 2004, 2007). Similarly, PPO suppression in dandelion *Taraxacum officinale* increased plant susceptibility to *P. syringae pv. tomato* (Richter et al., 2012). In *Taraxacum kok-saghyz,* PPO suppression increased fluidity of latex and resulted in collection of four- to fivefold more latex, indicating a role for lacticifer-specific PPO in wound sealing (Wahler et al., 2009).

In walnut (*Juglans regia*), PPO suppression caused localized cell death in leaves, observed as spontaneous necrotic lesions (Araji et al., 2014). A comparison between leaf transcriptomes and metabolomes of wild-type (WT) and PPO-silenced walnut plants showed changes in tyrosine metabolism that support a role for PPO activity in secondary metabolism, and an indirect role in cell death. By contrast, in PPO-suppressed apple fruits (Arctic^TM^ Apple) there were significant increases in both total phenolics (twofold) and ascorbic acid (10-fold) and an increase in water-soluble oxygen radical absorbance capacity (threefold) over control cultivars (Carter, 2012).

During environmental (biotic and abiotic) stresses, generation of reactive oxygen species (ROS) can cause major oxidative damage unless carefully controlled by cellular antioxidants, such as tocopherols and phenols, glutathione and ascorbic acid, and antioxidant enzymes, superoxide dismutase and catalase (Gill and Tuteja, 2010). Production of ROS contributes to altered redox potential of cells and tissues, linking external stimuli with stress responses. ROS production is also involved in control of growth and development at the organ, tissue, cellular, and subcellular levels (Kocsy et al., 2013). Antioxidants, synthesized in response to ROS, could maintain plant defenses against biotic and abiotic stresses and govern normal plant metabolism and morphological development.

In symbiosis, ROS and antioxidants play defensive roles during rhizobial invasion (Glyan’ko et al., 2007) and antioxidants are crucial for generating the low oxygen environment necessary for N_2_-fixation (Becana et al., 2000, 2010; Glyan’ko and Vasil’eva, 2010). To date, the antioxidant pathway has been investigated by overexpressing SOD in *Medicago sativa* (Rubio et al., 2001) and overexpressing glutathione synthase and homoglutathione synthetase in *Medicago truncatula* (Frendo et al., 2005), but overexpressing and silencing approaches have not previously been applied to PPO and the role, if any, of PPO in oxygen scavenging or redox reactions during normal plant physiology, growth and development of nodules is still to be elucidated.

Nodules of a range of legumes, with determinate (Chakraborty and Mandal, 2008; Mandal et al., 2009; Bargaz et al., 2013; Webb et al., 2013) or indeterminate (Datta and Basu, 1998; Glyan’ko et al., 2007; Webb et al., 2013) growth morphologies have measurable PPO activity. In the indeterminate nodules of red clover, PPO activity is high and is detected in specific cells and tissues (Webb et al., 2013). At least three classes of PPO enzymes reside in nodules, making the nodule a complex environment to study: the host plant itself produces a laccase (EC 1.10.3.2) as well as an enzyme with tyrosinase and catechol oxidase activities (EC 1.10.3.1), while free-living rhizobia also have tyrosinase activity (EC 1.14.18.1). Is the presence of plant PPO in nodules simply a localized defensive response to invasion and colonization by rhizobium bacteria (Glyan’ko et al., 2007) or has PPO activity an additional function?

While red clover (*Trifolium pratense* L.) and white clover (*Trifolium repens* L.) have PPO activity, activity is higher in both leaves and nodules of red clover (Webb et al., 2013). Recently published drafts of the red clover genome (de Vega-Bartol et al., 2014; Istvánek et al., 2014) will permit further analysis of the differentially expressed PPO genes of the large gene family (Winters et al., 2009; Webb et al., 2013). Transgenic plants with suppressed PPO activity (Sullivan and Hatfield, 2006; Lee et al., 2010) offer a unique opportunity to investigate the impact of reduced PPO on normal growth and development under a controlled environment, and especially on development and functioning of N_2_-fixing root nodules.

In indeterminate nodules of red clover, PPO activity is detected in specific cells and tissues (Webb et al., 2013). For example, intracellular PPO activity is co-localized with PPO substrates in the differentiating squashed cell layer (SCL). This region lies between the actively dividing nodule meristem and the fully developed SCL (Webb et al., 2013), which acts as an O_2_ diffusion barrier and is instrumental in maintaining anaerobic conditions. Taking into consideration that PPO belongs to the oxidoreductase class of enzymes, what effect does lack of plant PPO have on the nodule’s redox potential, normal nodule development and ultimately on N_2_-fixation?

## MATERIALS AND METHODS

### PLANT MATERIALS

#### RNAi PPO silenced red clover population

A segregating population of red clover (*T. pratense* L.) WT and PPO silenced plants was generated by crossing a WT clone with normal foliar PPO activity derived from WI-2 red clover germplasm (Sullivan et al., 2004) and an RNAi PPO transformant with undetectable levels of foliar PPO activity, NRC27-64-1 (Sullivan and Hatfield, 2006). Plants from 14 seeds resulting from the cross (designated RC1–RC14) were established in soil (Lee et al., 2010) and clones were taken from these plants and used for studies described below.

Clonal cuttings were taken from these 14 established plants. Actively growing shoots (5 cm × 4 cm shoots from each plant) were cut from each plant and stripped of expanded leaves. Cut stems were wetted, dipped in commercial hormone powder and planted in 1 L pots of medium grade vermiculite with N-free nutrient solution (Ryle et al., 1978) under a controlled environment of 16 h light, 25/15°C day/night temperatures, 300 μmol m^-2^ sec^-1^. Cuttings were kept under high relative humidity for 7 days, gradually reduced to 70% relative humidity after 7–14 days. Twelve clones were successfully rooted; two clonal lines, RC2 and RC3, did not survive. The established plants were inoculated with *Rhizobium leguminosarum* and watered daily with N-free nutrients (Ryle et al., 1978). Nodulated stock plants were grown in 2.5 L pots and propagated by transplanting single shoots with roots; these N_2_-fixing clonal stock plants have been maintained since 2007.

Roots with nodules were harvested from clonal cuttings of WT RC11 and RNAi RC4 grown in an aerated hydroponic system with N-free nutrients (Ryle et al., 1986) and inoculated with *R. leguminosarum.* These young, nodulated roots permitted rapid harvest, and had pink, actively fixing nodules at a range of developmental stages.

#### Detection of CaMV35S transgene in clonal plants

DNA was extracted using Nucleon^TM^ PhytoPure^TM^ (Amersham Biosciences) from young fully expanded leaves (200 mg) from all twelve established clones, nine of which had previously been shown to contain a pHannibal-based gene silencing construct (Lee et al., 2010). Presence of the transformed gene in the plants was confirmed by standard polymerase chain reactions (PCRs) using two primer sets to detect the CaMV35S promoter (35S1 5′-GCTCCTACAAATGCCATCA-3′ and 35S2 5′-GATAGTGGGAT- TGTGCGTCA-3′ or 35SA 5′ AAGGGTCTTGCGAAGGATAG-3′ and 35SB 5′ AGTGGAAAAGGAAGGTGGCT-3′. Presence of actin was used as a positive control for PCR using primers (RC actin F1 5′-TGGGATGACATGGAAAAGATCTGGCA-3′ and RC actin R1 5′-AGATTGGCACAGTGTGACTCACACCATC-3′).

#### Plant tissues, growth and morphology

Fresh weights (FWs) of shoots and roots were taken after removal of all vermiculite from the roots; all plant material was oven dried at 80°C for 1–2 days until dry weights (DWs) were stable. Analyses of shoots and roots for N content were as described elsewhere (Webb et al., 2010).

Shoot fresh and dry weights were taken. Measurements of nodule length were taken along the length of young, developing, pink nodules with a single meristem from basal end to meristem using a graticule and binocular microscope x16 magnification. Nodules were measured from 3 clonal plants. At least 10 separate roots with nodules were randomly selected from the full root length of plants 6 weeks from establishment and either 100 or 200 nodules were measured nodules were randomly selected from roots removed from the actively growing part of the root system (lower 66%) of older plants 30 weeks from establishment, and at least 35 nodules were measured.

Plant leaves, roots, roots with nodules or nodules alone were harvested from each clonal plant grown in vermiculite: roots were washed clean, and roots and nodules harvested and stored on ice at 2–4°C before weighing. The term ‘roots’ describes three types of root: the main thick tap root, thinner lateral roots which emerge from the tap root and fine roots. Both lateral and fine roots were nodulated. Tissues were flash frozen in liquid nitrogen (N) and stored at -80°C until required for determination of gene presence by PCR or measurements of PPO activity, redox potential values or antioxidant levels. Equivalent samples were freeze-dried and milled for carbohydrate analyses.

### BIOCHEMICAL ANALYSES

#### PPO activity

Quantitative analysis to compare PPO activity in red clover tissues using methyl catechol as substrate has been described previously (Webb et al., 2013). Average PPO activity of leaves, tap roots, lateral roots, fine roots, and nodules from all 12 clones (*n* = 3) ± SEM was expressed on a FW basis due to the low protein content in some tissues.

#### Measurement of redox potential and general anti-oxidation capacity

***Redox potential.*** Measurements of redox potential values were made on either freshly harvested or frozen tissues. Freshly harvested samples were taken from leaves, roots and nodules from all 12 clones (three WT and nine RNAi). Single, fully expanded trifoliate leaves with pulvinus (3 cm leaf diameter) were cut into 2–4 mm squares. Tap and lateral roots (100 mg FW, as 1.0 mm lengths), fine roots (100 mg FW, as 10 mm lengths) and nodules (100 mg FW, whole unless bifurcated then cut into 1 mm slices) were washed and tissues placed in 2 mL Eppendorf tubes with 1 mL of boiled and cooled, reverse osmosis (RO) purified water. The baseline redox potential value of RO water was -193 mV. After equilibration at room temperature for 2 h (leaves) or 3 h (roots and nodules), air bubbles were removed by tapping for about 30 s and samples mixed for 20–30 s. Measurements were recorded after 5–10 min, when the readings were stable, using a Micro Oxidation-Reduction Electrode (ORP-146C. Lazar Research Laboratories Inc.) connected to 6230N pH/mv/temp meter (Jenco) and graphs were generated using ArroxDox TM Plotter. Six datasets were taken on separate days giving a total of 18 WT (6 × 3) and 54 RNAi plants (6 × 9) measurements. Redox potential values recorded for each sample were combined for all six datasets and presented as average ± SEM.

Frozen leaves harvested from 3 WT (11 replicates) and 4 RNAi plants (10 replicates), and nodules from 3 WT (seven replicates) and 5 RNAi plants (nine replicates) were processed to determine the redox potential value and general antioxidant status of tissues on the same sample. Frozen samples (200–300 mg) were ground in 2 mL Eppendorf tubes and boiling RO water (1 mL) added. All samples were boiled at 100°C for 5 min to deactivate enzymes, cooled on ice and centrifuged at 20,000 × *g*. The supernatant was dispensed into clean tubes for general antioxidant assay (see below) while cooled boiled RO water (200 μL) was added to the remaining homogenized tissue for measurement of redox potential value after 2–3 h (see above). Baseline redox potential value of RO water was -121 mV. The data were presented as average ± SEM.

***Antioxidant assays.*** Antioxidant properties of aqueous extracts of leaves and nodules were expressed as Trolox equivalents, comparing samples to Trolox calibration curves in the range of mM g^-1^ FW. Three assays were used to determine the antioxidant activity of the samples: the radical scavenging assays, diphenylpicrylhydrazyl (DPPH) and ABTS+, and the ferric reducing antioxidant power, FRAP, as described elsewhere (Hauck et al., 2014).

***Ascorbic acid and glutathione.*** Ascorbic acid and glutathione concentrations in roots with nodules (200 mg FW; *n* ≥ 5) from WT RC11 and RNAi RC4 were measured as described elsewhere (Doulis et al., 1997). Samples were ground in liquid N, extracted cold in 2 mL 6% (w/v) meta-phosphoric acid and centrifuged at 15,000 *g* at 4°C for 5 min. Supernatant (0.5 mL) was added to Hepes/KOH buffer at pH 7.0 (100 μL) and enough 5 mM K_2_CO_3_ was added to bring the pH to 4.0–5.0 for ascorbate (AsA) and reduced ascorbate (DHA) assays, and pH 6.0–7.0 for glutathione (GSH) and oxidized glutathione (GSSG) assays. Samples were centrifuged at 15,000 × *g* for 5 min and supernatant collected.

Ascorbate was measured after 20 min in 0.1 M sodium phosphate buffer pH 7.5 and 0.2 mM dithiothreitol. Both AsA and DHA were determined by measuring the decrease in A_265_ (extinction coefficient of 14.3 cm^-1^ mM^-1^) after the addition of 1 U ascorbate oxidase (Sigma) to 1 mL of the reaction mixture containing the plant extract in 100 mM potassium phosphate, pH 7.0. GSH was determined by spectrophotometer readings at A_412_nm after extract was added to 0.1 M sodium phosphate buffer pH 7.5 and 0.6 mM 5′, 5′ dithiobis-(2-nitrobenzoic acid). GSSG was similarly measured at A_412_ nm after addition of NADPH and 0.5 U glutathione reductase (Sigma), oxidized glutathione (GSSG) was calculated by subtracting the reading for GSH. Readings were calculated from a standard curve.

#### Carbohydrate analyses

Roots with nodules (5–7 g FW) from 4 clones of WT RC11 and RNAi RC4 plants were freeze-dried and ground to a fine powder. Samples (*n* = 3) were taken from each plant and each assay repeated three times. Total starch content was determined using total starch assay kit (Megazyme International Ireland Ltd.). Total water-soluble carbohydrate (sucrose, fructose and glucose) of extracts were determined by the anthrone method as described elsewhere (Farrar et al., 2012); samples from WT RC11 and RNAi RC4 plants were also analyzed by HPLC to determine sugar content (Hauck et al., 2014).

### MICROSCOPY

#### Light and transmission electron microscopy (TEM) combined with immunogold labeling and Raman microspectrometry

Plants WT RC11 and RNAi RC4 were chosen for more detailed microscopic analysis. This was based on their transgenic status and consistency of nodule PPO activities and redox potential values: both PPO activities and redox potential values were high in WT RC11 and low in RNAi RC4. Nodule samples for light microscopy, TEM ultramicroscopy with immunogold labeling, and Raman microspectrometry were prepared from 6 week old clonal plants, as described elsewhere (Webb et al., 2013). Sections were prepared and examined from 4 to 5 nodules of both WT and RNAi plants to ensure accurate representation.

Using the Leica LMD software measuring feature, measurements were made of the distance from the inner cortex to the SCL (WT, *n* = 181; RNAi, *n* = 308), the size of starch grains in amyloplasts (WT, *n* = 190; RNAi, *n* = 329) in cells of the inner cortex and the size of the spherical cells of the nodule cortex, adjacent to SCL (WT and RNAi, *n* = 20). The latter was estimated by measuring across the cells at two points and calculating the area.

#### Phenolic analyses and Raman microspectrometry

Phenolic substrate analysis was by reversed phase HPLC, as described for leaf (Winters et al., 2008b; Webb et al., 2013). Extracts were made from leaves (600 mg FW) and roots with nodules (200 mg FW) from each clone, WT (*n* = 3) RNAi (*n* = 9), and from excised nodules from RC11 and RC4 (100 mg; *n* = 2).

Multivariate Raman images were collected from sections mounted on 1 mm thick silicon slides without coverslips from 1400 cm^-1^ to 1800 cm^-1^. Spectra were acquired using a Renishaw InVia Raman spectrophotometer fitted with a Leica microscope, a Prior H101RNLB mapping stage and a 514 nm green laser (Modu-Laser^TM^, S. Centreville, UT; USA). Samples were analyzed by Renishaw’s Streamline^TM^ mapping technology to generate large multivariate images and at a higher level of resolution than could be achieved by standard point mapping approaches. Streamline images were collected using ×50 magnification objectives (Leica) and a step size of 1.1 μm. Typical exposure times were 8 s and measurements were binned by a factor of 3 or more to reduce the number of pixels. Mosaic images of entire nodules could be collected in 8–12 h. Raman spectra for point mapping were collected using full laser power and a step size of 1.0 μm, with a typical exposure time for each spectral measurement of 10 s. A map consisting of 50 × 50 measurements took approximately 12 h to acquire.

Aromatic compounds in the sections were visualized by their Raman emission at 1601 cm^-1^ (symmetric stretching of the aromatic ring) which is the most intense band for many phenolics including lignin (Sun et al., 2011).

### EXPERIMENTAL DESIGN AND STATISTICAL ANALYSIS

Statistical analysis was by ANOVA, Genstat 14th edition, using plant status (WT or RNAi) as fixed effect. Plants were randomized, and positions re-randomized every 2 weeks, within the controlled environment cabinet.

## RESULTS

### RNAi PPO TRANSFORMED RED CLOVER POPULATION

A total of 12 separate clonal lines taken from plants (RC1–RC14) of segregating RNAi PPO transformed red clover population survived: one WT and one RNAi transformant died. Within these 12 clonal lines, three WT (RC1, RC11, and RC13) and nine RNAi plants were identified by genotype and phenotype. In WT plants, CaMV35S promoter was not detected and leaves had significantly higher (*p* < 0.001) PPO activity; by contrast, in RNAi transformants, CaMV35S promoter was detected and leaf PPO activity was reduced 150-fold (**Table 1**). PPO activity in roots and nodules reflected the pattern observed in leaves: the same three WT plants had significantly higher PPO activity in both roots (*p* < 0.001) and nodules (*p* < 0.001); PPO activity in RNAi transformants was reduced 43-fold in roots and sixfold in nodules. While PPO activity in leaf and root tissues was consistent within WT and RNAi plants, in nodules there was more variability (**Table 1**).

**Table 1:** Detection of genes and total polyphenol oxidase (PPO) activity in wild type (WT) and RNAi plants.

	Transgenic status			Total PPO activity (Δ_420_ OD/g FW/min)		
Plantcode	35S	Actin	Status	Leaf	Root	Nodule
RC1	-ve	+ve	WT	132.1 ± 27.79	32.9 ± 31.42	119.2 ± 57.20
RC11	-ve	+ve	WT	303.6 ± 137.42	41.5 ± 24.03	104.8 ± 64.47
RC13	-ve	+ve	WT	209.4 ± 63.90	80.7 ± 13.42	47.3 ± 7.77
**Mean**	-ve	+ve	**WT**	**215.0**± **50.92**	**51.7**± **14.12**	**90.4**± **27.29**
						
RC4	+ve	+ve	RNAI	1.1 ± 0.53	0.8 ± 0.32	1.8 ± 0.41
RC5	+ve	+ve	RNAi	1.5 ± 1.15	1.4 ± 0.41	14.8 ± 2.64
RC6	+ve	+ve	RNAi	1.9 ± 1.32	1.2 ± 0.26	7.5 ± 4.68
RC7	+ve	+ve	RNAi	0.5 ± 0.19	1.2 ± 0.14	9.8 ± 6.35
RC8	+ve	+ve	RNAi	1.1 ± 0.26	0.8 ± 0.23	61.8 ± 28.71
RC9	+ve	+ve	RNAi	0.7 ± 0.62	1.1 ± 0.24	11.85 ± 8.42
RC10	+ve	+ve	RNAi	2.7 ± 1.36	1.0 ± 0.41	9.7 ± 3.36
RC12	+ve	+ve	RNAi	2.5 ± 1.26	1.5 ± 0.49	13.6 ± 5.87
RC14	+ve	+ve	RNAi	0.5 ± 0.33	1.3 ± 0.06	9.6 ± 6.24
**Mean**	+ve	+ve	**RNAi**	**1.4 ± 0.30**	**1.2 ± 0.10**	**15.5 ± 4.35**
				***	***	***

-ve gene not detected; +ve gene detected; PPO activity (*n* = 3); data presented with ±SEM; ***p < 0.001.

### REDOX POTENTIAL AND GENERAL ANTIOXIDANT STATUS IN WT AND RNAi PLANTS

The redox potential value of fresh leaves, roots (tap, lateral, and fine) and nodules was measured from all 12 plants (**Table 2A**). There was a large and significant difference in redox potential value of fresh tissues between WT and RNAi plants, especially in leaves (*p* < 0.001). In RNAi transformants, redox potential values of root tissues were generally higher than in leaves, with greater similarity between WT and RNAi plants. Redox potential values were significantly different in tap roots (*p* = 0.01), fine roots (*p* = 0.01) and nodules (*p* = 0.05) of RNAi plants, but there was no significant difference between lateral roots of WT and RNAi transformants.

**Table 2A:** Redox potential value in fresh tissues in WT and RNAi plants.

		Redox Potential (mV)				
Plant Code	Status	Leaf	Tap root	Lateral root	Fine root	Nodule
RC1	WT	191.5 ± 8.43	289.0 ± 8.74	138.3 ± 21.99	186.0 ± 7.57	119.3 ± 4.06
RC11	WT	144.0 ± 26.55	146.7 ± 7.88	171.0 ± 3.61	106.7 ± 6.17	157.3 ± 2.67
RC13	WT	161.5 ± 23.36	152.3 ± 1.86	163.0 ± 24.01	147.7 ± 2.03	148.0 ± 6.81
**Mean**	**WT**	**165.7 ± 12.34**	**196.0 ± 23.52**	**157.4 ± 10.66**	**146.8 ± 11.81**	**141.6 ± 6.21**
						
RC4	RNAi	3.2 ± 3.25	112.0 ± 26.66	130.0 ± 16.17	91.7 ± 2.33	98.7 ± 12.47
RC5	RNAi	-10.3 ± 7.81	208.0 ± 37.11	158.7 ± 7.31	54.7 ± 12.44	36.3 ± 28.49
RC6	RNAi	10.0 ± 9.02	139.7 ± 3.28	165.0 ± 11.55	119.0 ± 18.45	25.3 ± 16.02
RC7	RNAi	-7.0 ± 8.46	142.3 ± 19.78	119.0 ± 15.87	129.3 ± 11.26	227.3 ± 4.06
RC8	RNAi	-11.8 ± 7.36	96.7 ± 17.40	116.3 ± 19.15	81.7 ± 15.56	93.0 ± 16.44
RC9	RNAi	22.3 ± 15.46	114.7 ± 9.06	138.0 ± 0.58	108.3 ± 7.69	56.7 ± 15.77
RC10	RNAi	5.0 ± 14.76	134.0 ± 12.50	163.0 ± 4.36	133.3 ± 6.01	71.7 ± 22.21
RC12	RNAi	-29.3 ± 16.47	113.3 ± 5.36	143.0 ± 3.21	129.7 ± 8.21	36.0 ± 23.00
RC14	RNAi	5.5 ± 13.48	128.0 ± 5.29	153.3 ± 2.19	118.7 ± 5.78	101.3 ± 7.84
**Mean**	**RNAi**	**–1.4 ± 4.03**	**132.1 ± 7.85**	**142.9 ± 4.59**	**107.4 ± 5.75**	**82.9 ± 12.36**
		***	**	**ns**	**	*

Leaf (*n* = 6), root tissues and nodules (*n* = 3); data presented with ± SEM; *p < 0.05; **p < 0.01; ***p < 0.001; ns, not significant.

Redox potential values and general antioxidant status of total cellular contents were also measured in extracts of frozen leaves and nodules from at least three clones of WT and RNAi plants (**Table 2B**). The clones used depended on tissue availability. Redox potential values in frozen tissues (**Table 2B**) were lower in RNAi than WT plants, as was previously observed for fresh tissues (**Table 2A**). No significant difference in redox potential values was observed for freshly harvested versus frozen WT leaves (**Tables 2A,B**). However, in frozen RNAi leaves, the redox potential value was significantly higher than in fresh leaf tissue (41.4 mV compared to 1.4 mV). In nodules the redox potential values were lower for frozen as compared to fresh tissues (3.7 mV versus 82.9 mV) and there was no significant difference in redox potential values between frozen nodules from WT and RNAi plants (WT 15.9 mV and RNAi 3.7 mV).

**Table 2B:** Redox potential values and antioxidant capacity of frozen leaves and nodules of WT and RNAi plants.

			Antioxidant capacity (Trolox equivalents mM g^-1^ FW)					
	Redox potential value (mV)		DPPH		ABTS		FRAP	
Status	Leaves	Nodules	Leaves	Nodules	Leaves	Nodules	Leaves	Nodules
WT	165.4 ± 5.82	15.9 ± 4.88	3.4 ± 0.22	0.4 ± 0.07	2.7 ± 0.38	0.4 ± 0.06	3.9 ± 0.51	0.3 ± 0.04
RNAi	41.4 ± 5.24	3.7 ± 3.93	3.8 ± 0.43	0.3 ± 0.04	3.7 ± 0.39	0.4 ± 0.04	5.1 ± 0.37	0.2 ± 0.03
	***	ns	ns	ns	ns	ns	ns	ns

Leaf (*n* ≥ 10), nodule (*n* ≥ 7); data presented with ±SEM; ***p ≤ 0.001; ns, not significant.

All three antioxidant assays gave similar results, with higher general antioxidant capacity in leaves than in nodules in both WT and RNAi plants, ranging from 2.65–5.11 Trolox Equivalents mM g^-1^ FW in leaves to 0.22–0.41 Trolox equivalents mM g^-1^ FW in nodules. Although antioxidant capacity tended to be higher in leaves of RNAi plants (**Table 2B**), there was no significant difference between the analyzed tissues of WT and RNAi plants.

### NATURAL PPO SUBSTRATE CONCENTRATIONS

Young, fully expanded leaves of both WT and RNAi plants contained *o*-diphenols, phaselic acid, caffeoyl DOPA (clovamide) and coumaroyl DOPA, and the monophenol coumaroyl malate (**Table 3A**). Total leaf phenolic concentration was slightly higher in RNAi transformants (2.85 ± 0.256 μmol/g FW) than in WT plants (2.01 ± 0.323 μmol/g FW), due to increased concentrations of coumaroyl DOPA (threefold higher) and clovamide (twofold higher) in RNAi plants. Only clovamide concentrations were significantly different (*p* = 0.018; **Table 3A**). Phaselic acid: clovamide ratio in leaves of RNAi plants was 1.68 ± 0.169 as opposed to 4.01 ± 1.33 in WT plants (*p* = 0.011).

**Table 3A:** Concentration of soluble phenolics in leaves of WT and RNAi plants.

		Concentration (μ mol/g FW)				
Plant code	Status	Caffeoyl malate (phaselic acid)^a^	Caffeoyl DOPA (clovamide)^a^	Coumaroyl malate^b^	Coumaroyl DOPA^a,b^	Total phenolics
RC1	WT	1.16	0.18	0.03	0.001	1.368
RC11	WT	1.80	0.49	0.07	0.022	2.388
RC13	WT	1.46	0.77	0.038	0.013	2.276
**Mean**	**WT**	**1.48 ± 0.185**	**0.48 ± 0.171**	**0.04 ± 0.016**	**0.012 ± 0.0061**	**2.01 ± 0.323**
						
RC4	RNAi	1.25	1.19	0.03	0.044	2.518
RC5	RNAi	1.56	1.42	0.06	0.076	3.111
RC6	RNAi	2.01	1.18	0.02	0.003	3.221
RC7	RNAi	1.03	0.51	0.02	0.018	1.574
RC8	RNAi	1.69	1.18	0.04	0.008	2.919
RC9	RNAi	1.21	0.90	0.04	0.018	2.172
RC10	RNAi	2.56	1.39	0.13	0.070	4.154
RC12	RNAi	2.26	1.13	0.08	0.076	3.541
RC14	RNAi	1.75	0.67	0.03	0.020	2.471
**Mean**	**RNAi**	**1.70 ± 0.170**	**1.06 ± 0.104**	**0.05 ± 0.012**	**0.037 ± 0.0100**	**2.85 ± 0.256**
		ns	*	ns	ns	ns

^a^PPO (catecholase) *o*-diphenol substrates; ^b^cresolase, potential monophenol substrate; WT (*n* = 3), RNAi (*n* = 9); data presented with ± SEM; *p < 0.05; ns, not significant.

Total soluble phenolic content of roots with nodules were about 10-fold lower than in leaves (**Tables 3A,B**; note concentrations are presented as nmol/g FW) Although the concentration of total soluble phenolics was higher in roots with nodules of RNAi transformants (343.7 ± 70.46 nmol/g FW) than in WT plants (220.5 ± 69.55 nmol/g FW), they did not differ significantly (**Table 3B**). Coumaroyl-tyrosine, clovamide and feruloyl-DOPA were the main constituents and these compounds varied considerably within samples, as reflected in high standard errors. Clovamide and feruloyl DOPA were about twofold higher in RNAi than WT plants (**Table 3B**). A further comparison of isolated nodules from clones of two representative WT and RNAi plants (RC11 and RC4) showed a similar trend with increased clovamide content in RNAi plants (**Table 5A**).

**Table 3B:** Concentration of soluble phenolics in roots with nodules of WT and RNAi plants.

		Concentration (n mol/g FW)			
Plant code	Status	Coumaroyl-tyrosine	Caffeoyl-DOPA (clovamide)^a^	Feruloyl-DOPA^a^	Total phenolics
RC1	WT	35.5	51.9	8.8	96.2
RC11	WT	105.9	116.5	6.2	228.7
RC13	WT	198.3	123.8	14.6	336.7
**Mean**	**WT**	**113.2 ± 47.14**	**97.4 ± 22.87**	**9.9 ± 2.46**	**220.5 ± 69.55**
					
RC4	RNAi	136.3	59.2	3.6	199.2
RC5	RNAi	52.9	194.1	26.4	273.4
RC6	RNAi	168.6	276.9	30.1	475.6
RC7	RNAi	170.4	172.4	15.8	358.6
RC8	RNAi	104.5	55.8	18.3	178.6
RC9	RNAi	14.0	424.8	21.6	460.4
RC10	RNAi	114.7	43.9	11.2	169.9
RC12	RNAi	82.1	85.4	4.2	171.7
RC14	RNAi	170.5	619.7	15.4	805.5
**Mean**	**RNAi**	**112.7 ± 18.51**	**214.7 ± 65.57**	**16.3 ± 3.02**	**343.7 ± 70.46**
		ns	ns	ns	ns

*^a^Potential PPO substrate; WT (*n* = 3), RNAi (*n* = 9); data presented with ± SEM; ns, not significant.*

### SHOOT RE-GROWTH AND N CONTENT

While under controlled environment, plants were regularly cut back and shoot re-growth harvested after 14 days. Data was combined from different clones taken from all 12 plants harvested separately over 3 years; these data showed that both shoot re-growth and N content were lower in RNAi transformants in all three harvests. Shoot re-growth was significantly lower in one harvest and in the harvest average (**Table 4A**).

**Table 4A:** Plant phenotype: shoot dry weight (DW) and N content of shoot re-growth of clonal plants over 14 days taken from different clonally propagated WT and RNAi plants in separate harvests over 3 years.

	Harvests after 14 days shoot re-growth							
Plant status	Harvest 1		Harvest 2		Harvest 3		Average	
	Dry wt (g)	N (%)	Dry wt (g)	N (%)	Dry wt (g)	N (%)	Dry wt (g)	N (%)
WT	1.01 ± 0.148	5.50 ± 0.108	2.75 ± 0.090	4.87 ± 0.062	2.84 ± 0.134	4.03 ± 0.118	2.43 ± 0.155	4.89 ± 0.093
RNAi	0.83 ± 0.063	5.45 ± 0.150	1.91 ± 0.102	4.76 ± 0.049	2.77 ± 0.350	3.83 ± 0.140	1.79 ± 0.102	4.78 ± 0.068
	ns	ns	***	ns	ns	ns	**	ns

WT (*n* = 27), RNAi (*n* = 81); data presented with ±SEM; **p < 0.01; ***p < 0.001; ns, not significant.

In the harvests chosen to examine nodules closely, there was no significant difference in either re-growth or N status of the shoots. However, WT and RNAi transformant nodules differed markedly, developing RNAi transformant nodules were longer (**Table 4B**) and initially appeared to form fewer nodule meristems (i.e., were less bifurcated) though older nodules appeared to develop multiple meristems, creating almost triangular nodules.

**Table 4B:** Plant phenotype: shoot DW after 14 days re-growth, N content of leaf and root nodule length in 6 week old clonal cuttings of WT and RNAi plants.

Plant code	Status	Shoot DW (g)	N content leaf (%)	Nodule length (mm)
RC1	WT	2.91 ± 0.61	3.79 ± 0.111	0.985 ± 0.0189**^a^**
RC11	WT	2.58 ± 0.38	4.18 ± 0.048	0.884 ± 0.0290**^b^**
RC13	WT	3.03 ± 0.45	4.11 ± 0.158	0.871 ± 0.0236**^b^**
**Mean**	**WT**	**2.84 ± 0.134**	**4.03 ± 0.118**	**0.940 ± 0.0161**
				
RC4	RNAi	2.45 ± 0.246	4.04 ± 0.127^d^	1.052 ± 0.0356
RC5	RNAi	3.15 ± 0.322	4.03 ± 0.201	1.160 ± 0.0525
RC6	RNAi	1.02 ± 0.158	3.44 ± 0.092^d^	1.301 ± 0.0570
RC7	RNAi	2.08 ± 0.067	3.04 ± 0.221	1.164 ± 0.0446
RC8	RNAi	3.82 ± 0.500	4.02 ± 0.172	1.196 ± 0.0425
RC9	RNAi	2.40 ± 0.193	4.07 ± 0.059	1.029 ± 0.0325
RC10	RNAi	3.80 ± 0.303	3.96 ± 0.103	1.093 ± 0.0423
RC12	RNAi	4.24 ± 0.789	4.39 ± 0.121	1.149 ± 0.0407
RC14	RNAi	2.01 ± 1.103**^c^**	3.47 ± 0.277	1.152 ± 0.0431
**Mean**	**RNAi**	**2.77 ± 0.332**	**3.83 ± 0.140**	**1.144 ± 0.0148**
		ns	ns	***

Shoot DW and N content (*n* = 3 with ± SEM; ^c,d^*n* = 2 with ±SD); nodules (*n* = 100 or ^a^*n* = 400; ^b^*n* = 200) WT and RNAi means and SEM’s are based 3 and 9 df; ***p < 0.001; ns, not significant.

This morphological difference in nodule length between WT and RNAi plants was consistent and was observed in two other experiments: newest root growth of a complete set of older clonal plants (**Table 4C**) and in two contrasting phenotypes WT RC11 and RNAi RC4 (**Table 5A**).

**Table 4C:** Plant phenotype: shoot DW after 14 days re-growth and root nodule length in lower half of root systems of 30 week old, established WT and RNAi plants.

Plant code	Status	Shoot DW(g)	Nodule length (mm)
RC1	WT	0.86 ± 0.132	3.07 ± 0.202
RC11	WT	1.48 ± 0.121	1.80 ± 0.104
RC13	WT	0.88 ± 0.083	4.31 ± 0.118
**Mean**	**WT**	**1.07 ± 0.205**	**2.96 ± 0.111**
			
RC4	RNAi	1.74 ± 0.505	1.97 ± 0.092
RC5^a^	RNAi	1.09 ± 0.515	2.48 ± 0.284
RC6	RNAi	0.65 ± 0.109	3.52 ± 0.143
RC7	RNAi	1.11 ± 0.472	2.85 ± 0.107
RC8	RNAi	1.30 ± 0.573	4.43 ± 0.101
RC9	RNAi	1.37 ± 0.297	2.56 ± 0.149
RC10	RNAi	1.61 ± 0.359	3.83 ± 0.111
RC12	RNAi	1.14 ± 0.212	4.34 ± 0.148
RC14	RNAi	0.95 ± 0.085	5.10 ± 0.110
**Mean**	**RNAi**	**1.22 ± 0.110**	**3.47 ± 0.055**
		ns	***

Shoot DW (*n* = 3); nodule length (*n* =≥ 60, except ^a^*n* = 35); data presented with ±SEM. WT and RNAi means and SEM’s are based 3 and 9 df; ***p < 0.001; ns, not significant.

### ANALYSES OF CONTRASTING PHENOTYPES OF WT AND RNAi PLANTS

Shoot re-growth and N content of WT and RNAi plants were similar. However, morphology of young developing nodules of all independent RNAi transformants differed from WT plants. Three independent RNAi transformants (RC4, RC8, and RC10) were studied at the light and ultramicrosopic levels and were found to differ from the three WT plants. Normal initiation and development of many nodules of WT red clover plants has been observed and published elsewhere (Webb et al., 2013). The phenotype of nodules of RNAi plants proved remarkably consistent, with nodules of RNAi RC4 consistently presenting very low PPO activity. WT RC11 and RNAi RC4 plants are representative of the overall phenotypes. Contrasting phenotypes WT RC11 and RNAi RC4 were used for preliminary biochemical and chemical analyses.

Nodules of RNAi RC4 were longer than WT RC11 (*p* < 0.001) and contained more clovamide (**Table 5A**). While total non-structural carbohydrate and water-soluble carbohydrate of nodulated roots of RC4 and RC11 did not differ significantly, RC4 contained significantly less starch (*p* < 0.01) and more non-reducing sugar, sucrose (*p* < 0.05) than RC11 (**Table 5A**). The reducing sugars were identified and confirmed by HPLC as mainly glucose, with virtually no fructose present in the samples.

**Table 5A:** Morphology, chemistry, and biochemistry of roots and nodules of two contrasting phenotypes, WT RC11 and RNAi RC4 plants.

		Nodules		Root with nodules				
Plant code	Status	Length(mm)	Caffeoyl DOPA (Clovamide)(n mol/g FW)	Starch(mg/g DW)	Reducing sugars(mg/g DW)	Non-reducing sugars(mg/g DW)	Total soluble carbohydrate (mg/g DW)	Total non-structural carbohydrate (mg/g DW)
RC11	WT	0.91 ± 0.026	52.64 ± 31.970	4.95 ± 0.262	41.74 ± 1.342	16.96 ± 1.333	59.03 ± 2.030	63.98 ± 2.061
RC4	RNAi	1.08 ± 0.024	674.01 ± 373.222	3.58 ± 0.285	39.98 ± 3.286	21.01 ± 1.316	62.35 ± 3.829	65.93 ± 3.355
		***	ns	**	ns	*	ns	ns

Samples taken from equivalent plants (16–20 weeks old); nodule length (*n* =≥ 268); clovamide (*n* = 3); starch and sugars in roots with nodules harvested from RC11 WT and RC4 RNAi (*n* = 12; 4 clonal plants and 3 reps); data presented with ± SEM; *p < 0.05; **p < 0.01; ***p < 0.001; ns, not significant.

A preliminary biochemical analysis was made on excised roots with nodules remaining from harvests from RNAi RC4 and WT RC11. These data indicated that total concentration of antioxidants, glutathione, and ascorbate, were similar and the ratio of reduced to oxidized ascorbic acid and glutathione did not differ significantly between the WT and RNAi plants (**Table 5B**).

**Table 5B:** Ascorbic acid and glutathione content in roots with nodules of two contrasting phenotypes, WT RC11 and RNAi RC4 plants.

Plant code	Total AsA (μM g^-1^ FW)	AsA (μM g^-1^ FW)	DHA (μM g^-1^ FW)	DHA:AsA	Total GSH (μM g^-1^ FW)	GSH (μM g^-1^ FW)	GSSG (μM g^-1^ FW)	GSSG:GSH
WT	34.7 ± 5.32	24.3 ± 3.52	10.4 ± 3.43	0.5 ± 0.15	1147.5 ± 146.19	225.8 ± 25.49	921.7 ± 129.11	4.1 ± 0.52
RNAi	31.2 ± 4.66	20.8 ± 3.46	10.4 ± 2.69	0.6 ± 0.15	1457.3 ± 261.17	231.3 ± 23.39	1244.0 ± 266.05	5.7 ± 1.43
	ns	ns	ns	ns	ns	ns	ns	ns

*AsA (*n* = 6), GSH (*n* = 5); data presented with ±SEM; ns, not significant; total AsA, ascorbic acid and dehydroascorbic; AsA, ascorbic acid; DHA, dihydro ascorbic acid; total GSH, glutathione and glutathione disulphide; GSH glutathione; GSSG glutathione disulphide.*

### RAMAN MICROSPECTROMETRY, LIGHT AND TEM MICROSCOPY OF WT RC11 AND RNAi RC4

Nodules from two representative clonal plants, WT RC11 and RNAi RC4, which showed typical anatomical changes in their anatomy under light microscopy and TEM, were also prepared for sectioning for visualizing by Raman microspectrometry. The region of focus for TEM was the developing SCL, as described previously (Webb et al., 2013).

**FIGURE 1:**
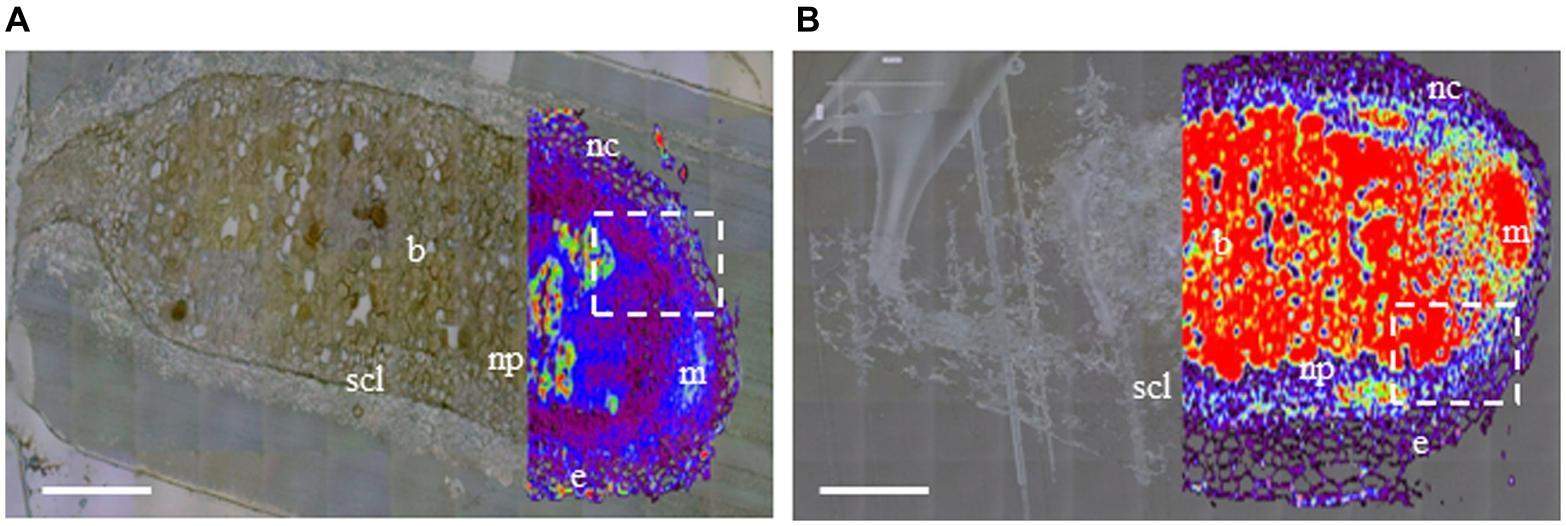
**Distribution of phenolics in nodule sections of two contrasting polyphenol oxidase (PPO) phenotypes, wild-type (WT) RC11 and RNAi RC4 plants as visualized by Raman microspectrometry overlaying an image generated by white reflected light.** Raman images were collected using a 514 nm laser from 1400 to 1800 cm^-1^ and over-laid the same image collected by reflected white light in unstained ultrathin sections of two nodules in longitudinal section **(A)** WT RC11 and **(B)** RNAi RC4. The nodule meristem (m), epidermis (e), nodule cortex (nc), nodule parenchyma (np) and N_2_-fixing bacteroids (b) are clearly visible, along with the fully developed squashed cell layer (SCL). The white, dashed rectangles indicate the regions either side of the meristem where the SCL begins to differentiate; this region in another nodule is analyzed further by transmission electron microscopy (TEM) in **Figure 2**. The colors represent concentration of phenolics (as visualized by Raman emission at 1601 cm^-1^), from transparent equating with low levels of phenolics through black – purple – blue – white – green – yellow – orange – to red equating with high levels of phenolics. Bars = 200 μm.

Raman microspectrometry showed a different pattern of phenolic distribution in six WT and six RNAi nodules. Representative images of whole nodule sections from each clone are shown in **Figures 1A,B**. In WT RC11, SCL was as previously described (Webb et al., 2013), with a generally low phenolic signal within the nodule, and with discrete, localized stronger signal apparent in epidermal cells, in nodule cortex outside the SCL and within the infected zone containing bacteroids (**Figure 1A**). In RNAi RC4, generally higher levels of phenolics were distributed through the nodule, accumulated at higher levels in the regions of the plant meristem and near bacteroids (**Figure 1B**). The developing or developed SCL was less obvious in nodules of RNAi RC4 than WT RC11.

**FIGURE 2:**
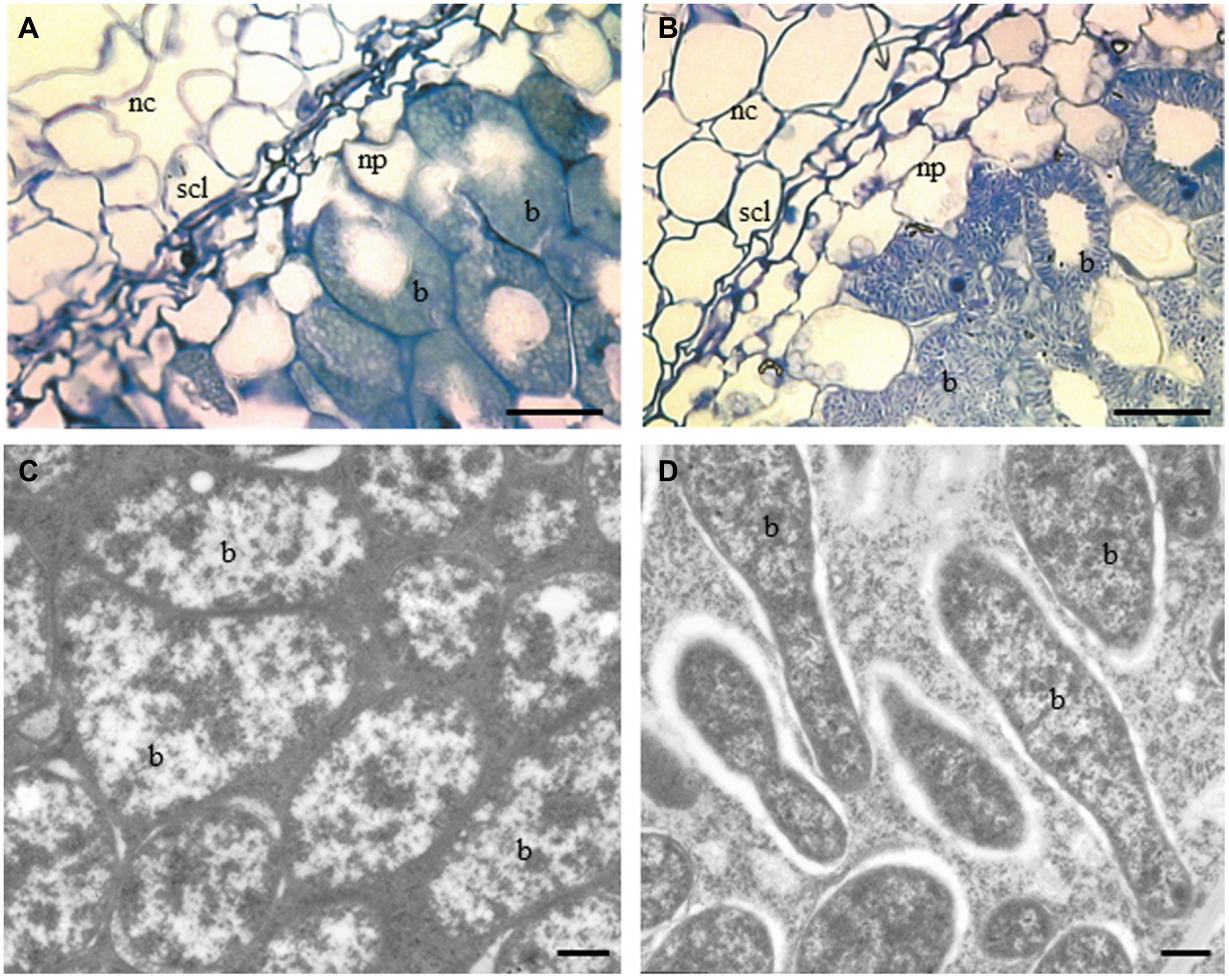
**Light and TEM micrographs of nodules of sections of two contrasting PPO phenotypes, WT RC11 and RNAi RC4 plants. (A)** and **(B)**, Light micrographs showing nodule cortex (nc), nodule parenchyma (np), SCL and bacteroids (b). **(A)** WT RC11 nodules had 3–4 celled SCL and rounded bacteroids. Average width cells of SCL = 37.53 ± 0.860 μm, average area cells nc = 493.7 ± 25.62 μm^2^. B, RNAi RC4 nodules showing wider 5–6 celled SCL, which are less squashed and, more granular bacteroids (b). Average width cells of SCL = 60.05 ± 1.079 μm, average area cells nc = 1164.8 ± 117.32 μm^2^. **(C,D)** TEM micrographs showing bacteroids within nodules (b). **(C)** WT RC11 rounded tightly packed bacteroids. **(D)** RNAi RC4 elongated granular bacteroids (b). Bars **(A,B)** = 25 μm, **(C,D)** = 500 nm.

At the cellular level, changes were detected in sections examined under a light microscope. These images showed that there were significant differences between WT RC11 and RNAi RC4. The fully developed SCL in WT RC11 was significantly (*p* < 0.001) narrower than in the RNAi transformant RC4 (37.53 ± 0.860 μm and 60.05 ± 1.079 μm respectively) and the SCL contained fewer cells. WT RC11 had 3–4 cells in the SCL as opposed to 5–6 cells in RNAi RC4 that are not actually squashed (**Figures 2A,B** respectively). Measurements of the spherical cells of the nodule cortex indicated that in RNAi RC4 cells were 236% larger (*p* < 0.001) than those of WT RC11 (1164.8 ± 117.32 μm^2^ versus 493.7 ± 25.62 μm^2^).

Under TEM, bacteroids in WT RC11 nodules appeared normal, densely packed and rod shaped (**Figures 2A,C**), but in RNAi RC4 the bacteroids were more granular with large intercellular spaces between peribacteroid membrane and rhizobium (**Figures 2B,D**) with some immunogold PPO label in spaces. Starch-filled amyloplasts were more numerous and the starch grains significantly larger (*p* < 0.001) within the inner cortex of RNAi RC4, measuring 8.13 ± 0.091 μm as opposed to 6.07 ± 0.098 μm in WT RC11 (not shown).

## DISCUSSION

### PPO ACTIVITY, GROWTH, REDOX POTENTIAL VALUES AND PHENOLIC CONTENT OF WT AND RNAi AERIAL TISSUES

Wild-type and RNAi transformants of red clover survived as N_2_-fixing plants for more than 6 years as clonally propagated cuttings growing under controlled environmental conditions indicating competent N_2_-fixation. Shoot and leaf N content and re-growth were used as simple parameters to indicate biological N_2_-fixation (Hardarson and Danso, 1993; Azam and Farooq, 2003). WT and RNAi plants grew similarly, with similar total N content (**Tables 4A,B**), although at harvest 2, shoot regrowth of RNAi transformants was significantly lower (*p* < 0.001) than in WT plants (**Table 4A**); this may have resulted from amplified differences in initial size and number of growing buds of the clones.

Overall, both PPO activity and redox potential values in leaves of RNAi transformants were lower than in WT plants (**Tables 1 and 2A**). This lower redox potential value in leaves of RNAi plants suggested a significant change in redox homeostasis in these plants lacking PPO, which created a more reduced environment within the leaves. Despite this altered redox status, growth of RNAi plants was similar to WT plants under optimal, non-stressed conditions.

The population of WT and RNAi red clover plants was created by crossing a single RNAi transformant (Sullivan and Hatfield, 2006) with a WT plant, derived from a population of WI-2 germplasm (Sullivan et al., 2004). Therefore, it is possible that a gene affecting redox potential values could have co-segregated with the RNAi PPO insert. Segregation from the original cross is skewed in favor of plants containing the RNAi transgene insert (9:3), suggesting more than one locus in the parental RNAi plant. This makes co-segregation of the silencing transgene and a gene affecting redox potential values less likely. High leaf PPO activity and high redox potential values were correlated in WT plants, and low leaf PPO activity and low redox potential values in RNAi transformants (**Tables 1 and 2A**), suggesting that changes observed in redox potential values are due to silenced or reduced PPO enzyme activity.

This conclusion was further supported by measurements of redox potential values from leaves of glasshouse-grown WT plants and a low leaf PPO genetic mutant (Aa4512) showing 26-fold reduction in leaf PPO (Winters et al., 2008b). While leaf redox potential data from WT plants grown in a controlled environment or glasshouse were similar at 165.7 mV and 122 mV respectively, leaf redox potential value of the PPO mutant was 22 mV, closer to data from RNAi transformants (-1 mV) than to leaves from WT plants in either growth environment.

Red clover leaves contain known natural substrates of PPO, phaselic acid and clovamide, (Winters et al., 2008b; Webb et al., 2013). Significantly, higher levels of phenols accumulated in leaves of RNAi plants (**Table 3A**), mainly due to increased concentrations of clovamide and coumaroyl DOPA. Transformants of apple (Arctic Apple), created using suppressing transgenes of PPO also showed increased total phenolics (twofold), along with high levels of ascorbic acid (10-fold), and a higher water-soluble oxygen radical absorbance capacity (threefold) than found in control cultivars (Carter, 2012). In the absence of PPO enzyme activity, potential phenolic substrates in red clover leaves could accumulate.

Growing clonal plants under controlled conditions reduced environmental and genetic variation; however, data for leaf redox potential values and phenolic content were variable. This may reflect natural variation in developmental stages or leaf position: leaves were harvested as young but fully expanded leaves and were expected to be at approximately the same developmental stage, but could be exposed to different light levels.

Interestingly, repeating measurements of redox potential values on frozen leaves highlighted potential issues with harvest and storage of these samples (**Tables 2A,B**). Fresh and frozen WT leaves had the same redox potential value of 165 mV, indicating an identical redox state. By contrast, redox potential values for fresh RNAi leaves was -1.4 mV and frozen leaves 41.4 mV, indicating that freezing RNAi leaves had altered the reducing environment. A break-down in cell compartmentation in frozen cells should have permitted release of all redox active compounds, which may not have occurred with fresh tissue. There may have been a greater contribution from oxidized compounds released from subcellular components in frozen extracts.

### PPO ACTIVITY, REDOX POTENTIAL VALUES, ANTIOXIDANT CAPACITY, MORPHOLOGY AND ANATOMY OF WT AND RNAi NODULES

In WT plants, PPO activity in nodules was lower than in leaves, as previously described (Webb et al., 2013). Variation within replicate samples was generally very high: roots with nodules, or nodules alone, were harvested regardless of developmental stage and may explain some of the data variation observed in these tissues. Roots with nodules have different ratios of root: nodule and young, developing nodules will be highly represented, whereas older, more fully developed, nodules with a senescent zone may be harvested individually. In addition, storing nodule samples on ice prior to flash freezing could cause changes to concentrations of labile metabolites.

Significant variation was observed in PPO activity and redox potential values between nodule samples. In RNAi RC8, nodule PPO activity was higher than in WT RC13 plant (**Table 1**), while nodule redox potential value of RC7 was higher than any WT plants (**Table 2A**). These extreme outlying samples only occurred in root systems and may be due to tyrosinase activity in bacteria and fungi such as *Arthobacter spp.* and *Gibberella zeae* (anamorph *Fusarium graminearum*), species which have previously been shown to contaminate nodules grown under similar conditions (Webb et al., 2010).

The relationship between PPO activity and redox potential values showed the same general trend in nodules as seen in leaves: RNAi nodules had lower PPO activity and lower redox potential values than WT nodules (**Table 2A**), indicating a more reduced environment in the absence of PPO. The same trend in redox potential values was observed in frozen nodules, although the readings were lower: both WT and RNAi frozen nodules (15.9 mV and 3.7 mV respectively; **Table 2B**) were less oxidized than fresh nodules (141.6 mV and 82.9 mV; **Table 2A**), which in contrast to leaf tissues may have been due to increased release of reduced species from subcellular compartments in frozen tissues.

As previously described for leaves, a comparison was made between nodules harvested from a population of glasshouse-grown WT and low leaf PPO genetic mutant (Aa4512) plants. The low leaf PPO phenotype is caused by a mutation in a single gene of the differentially expressed red clover PPO gene family. PPO activity in nodules of these low leaf PPO mutants was reduced 2–3 fold, but higher than detected in RNAi plants. However, average redox potential values of nodules from WT and low leaf PPO mutants did not differ significantly from each other (126 mV and 143 mV respectively), or from WT nodules from plants grown under controlled environment (142 mV; **Table 2A**).

Antioxidant capacity may be protected more carefully in nodules than in leaves since an anaerobic environment is required for successful N_2_-fixation; this may be achieved by employing a range of different mechanisms. RNAi root systems showed important changes in their morphology and chemistry. Young, developing RNAi nodules were on average longer than WT nodules (**Tables 4B,C**). Roots with nodules (**Table 3B**) and nodules alone (**Table 5**) had higher concentration of soluble phenolics, especially the known PPO substrate, clovamide. While the data were variable and not significantly different, Raman microspectrometry of several typical RNAi RC4 nodules showed major phenolic accumulation in the meristem, and within the SCL, particularly near the bacteroids in young developing nodules from RNAi RC4 as compared to WT RC11 (**Figures 1A,B**).

Further comparison of TEM sections of nodules of two contrasting clones RNAi RC4 and WT RC11 revealed that lack of PPO subtly altered nodules, altering normal development of the SCL (or oxygen diffusion barrier) and causing changes in morphology of the developing N_2_-fixing bacteroids (**Figures 2A–D**). Interestingly, nodule anatomy of genetic mutants was more similar to RNAi nodules: the SCL was wider (56.7 ± 1.117 μm; *n* = 183; *p* = 0.043) and starch grains of the inner cortex were larger than in WT nodules (9.027 ± 0.098 μm; *n* = 373). These data indicate that reduction in PPO activity affects normal nodule development and that localized PPO activity may be required for optimal development of the SCL.

In a previous study, PPO enzyme was detected by immunogold labeling in the cells of the developing SCL, between the apical meristem and the fully differentiated SCL, and was associated with starch grains. As the SCL developed and the cells became thickened and squashed, levels of labeled PPO enzyme and associated starch grains were reduced to very low levels (Webb et al., 2013). In the study described here, lack of PPO activity in the early stages of SCL formation appeared to alter cellular differentiation, producing more layers of larger, non-squashed cells, with less obviously thickened cell walls. The abnormal shape of the bacteroids may indicate early bacteroid senescence resulting from higher free oxygen concentrations within the nodules. In *Lotus japonicus*, suppressing symbiotic leghaemoglobin (LbRNAi) increased nodule number in LbRNAi plants and caused major changes to normal bacteroid differentiation within these determinate nodules (Ott et al., 2009). In the study described here, there appeared to be fewer nodules in WT than in RNAi plants (171.1 ± 66.12 and 247.1 ± 102.13 nodules/g DW respectively) suggesting that more nodules may have been initiated but failed to develop fully in RNAi plants.

Early nodule senescence is possibly caused by lack of PPO activity and resultant accumulation of excess phenolics in specific cells and tissues, which together fail to guarantee differentiation of an active oxygen diffusion barrier essential for establishment of N_2_-fixation. In the absence of sufficient fixed N, the host plant will continue to permit nodule initiation, and the plasticity of the plant antioxidant response could eventually result in the maturation of some larger fully functional N_2_-fixing nodules. A study in *M. sativa,* inoculated with catalase mutant *Sinorhizobium meliloti*, showed that reduced levels of catalase production during nodule formation resulted in bacteroids failing to differentiate and mature; this was followed by rapid senescence (Jamet et al., 2003). This indicates that elevated peroxide levels, a form of oxidative stress, inhibits formation of healthy bacteroids.

Lack of PPO resulted in differences in carbohydrate concentration and distribution in roots with nodules, with RNAi RC4 nodules containing less starch, possibly in larger starch grains, and more non-reducing sugar (as sucrose) than WT RC11 nodules. Indeterminate nodules of red clover and determinate nodules of soybean have similar carbohydrate composition (Streeter, 1980). Fructose concentration was very low in red clover nodules, suggesting relatively rapid utilization of fructose in nodules as previously reported in nodules of soybean (Streeter, 1980, 1981).

Polyphenol oxidase is associated with starch-accumulating plastids, chloroplasts in photo-synthetically active tissue (Sommer et al., 1994) and amyloplasts in storage organs (Stensballe et al., 2008). Chloroplastic starch is mobilized during the dark period while amyloplastic starch is stored until there is a high energy demand on the tissue or plant. During senescence, determinate nodules have been observed to lose their starch content (Puppo et al., 2005). The significantly lower starch content of RNAi RC4 roots with nodules may reflect a lower level of starch mobilization from leaves to the root systems, or a higher local metabolism. In nodules lacking PPO, an increased energy turnover may be required for the development of the SCL and to maintain biosynthesis of antioxidants.

Successful N_2_-fixing symbiosis depends on O_2_ depletion and maintenance of low O_2_ levels within nodules, with antioxidants of the plant ascorbic acid-gluthathione cycle playing a major role (Becana et al., 2000, 2010; Matamoros et al., 2003; Puppo et al., 2005). Elevated levels of antioxidants, such as ascorbate peroxidase, contribute to the oxygen scavenging capacity of the peripheral cell layers, forming an oxygen diffusion barrier in nodules of a range of legumes, including red clover (Dalton et al., 1998). Dalton also postulated that in addition to the oxygen barrier and leghemoglobin, that oxygen consumption at the endodermis could be a key component of the oxygen diffusion barrier. We have observed high levels of PPO close to the SCL layer which could provide an O_2_ depleting function in the presence of phenol substrates.

Preliminary data described here indicated no change in ascorbic acid or gluthathione content of nodules, though changes in redox potential of stored nodules could indicate changes in labile metabolites. *Rhizobium* bacteria may also have roles in developing the optimal environment for symbiosis. In *Rhizobium etli*, tyrosinase activity was demonstrated to protect invading symbionts against reactive oxygen species and phenolic compounds produced by the plant during early symbiosis (Cubo et al., 1997; Pinero et al., 2007) and *R. etli* and *R. fredii* both synthesize melanin in bacteroids (Cubo et al., 1997). There is also direct evidence of the role of bacterial glutathione in protecting nodules from reactive oxygen species, which may determine nodule senescence (Muglia et al., 2008).

In conclusion, all these data point to a major change in oxido-reduction status of plants lacking normal high level of PPO, resulting in destabilized redox pathways. Lack of PPO in red clover plants does not have an obvious effect on plants grown under optimal, stress-free N_2_-fixation conditions. However, the normal WT biochemistry, chemical composition, and developing morphology/anatomy of nodules were significantly altered.

Studies of the metabolome of leaves and nodules lacking PPO activity will determine any major changes in plant redox and secondary metabolism. Ultrastructural studies on the SCL would elucidate further any role PPO, and the resulting change in redox status, has in cellular differentiation and cell wall thickening, and its role in creating the barrier to oxygen diffusion in red clover nodules. Along with studies on specific antioxidants in N_2_-fixing nodules in PPO-free red clover, use of a rhizobium lacking PPO would clarify the roles of both plant and bacterial PPO in the initiation and successful development of legume-rhizobium symbiosis.

## Conflict of Interest Statement

The authors declare that the research was conducted in the absence of any commercial or financial relationships that could be construed as a potential conflict of interest.
